# Supplementary material for: High Atlastin 2-2 (ATL2-2) Expression Associates with Worse Prognosis in Estrogen-Receptor-Positive Breast Cancer
Source: Genes (Basel). 2023 Jul 29;14(8):1559. doi: 10.3390/genes14081559 (PMC10454310; doi:10.3390/genes14081559)
Supplement: Supplementary file 1 [file genes-14-01559-s001.zip › Supplement_Figures.pdf]

Supplement Figures

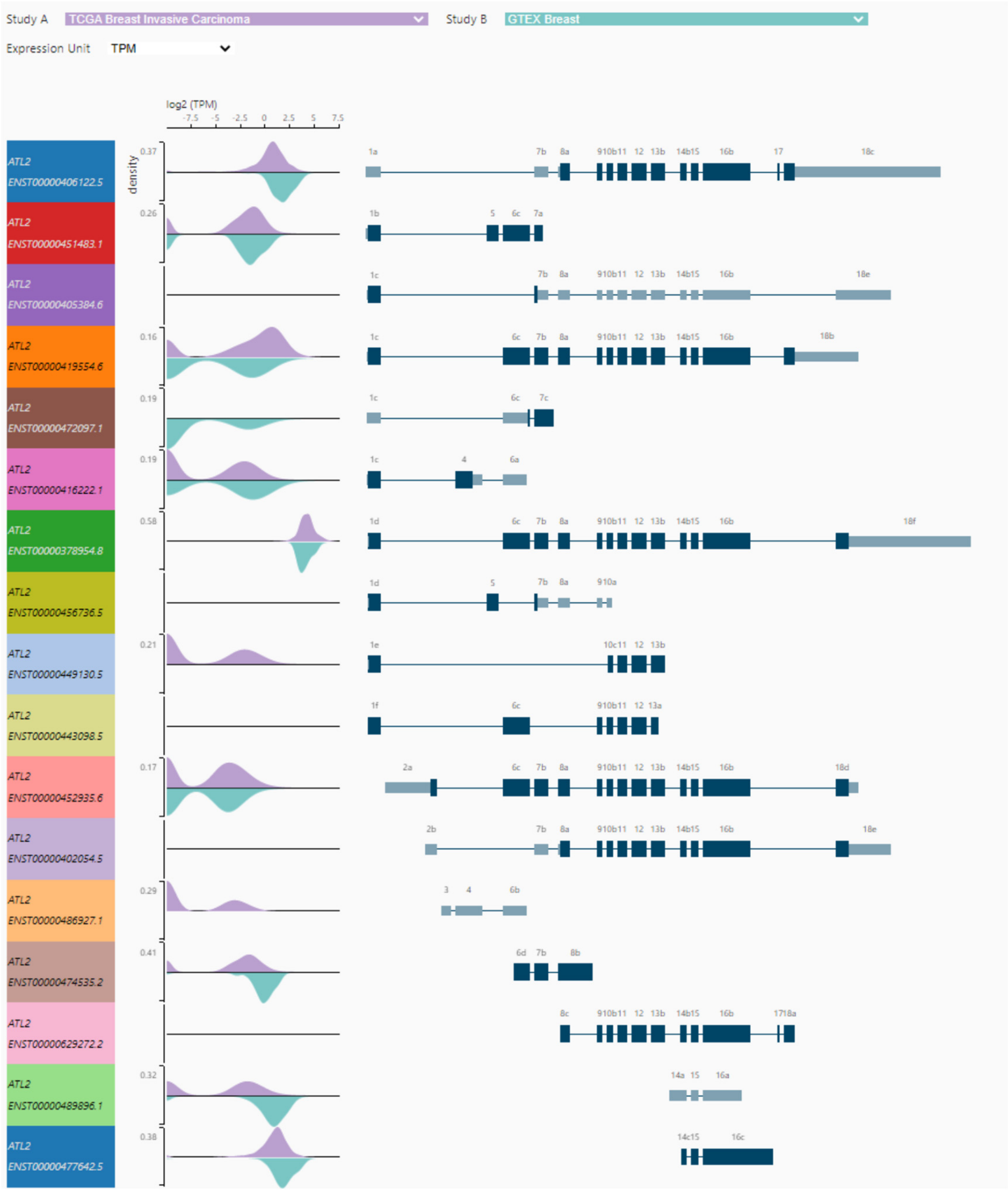

Figure S1. ATL2-2 transcript levels (ENST00000419554.6) are higher in TCGA breast tumors than in GTEX breast. Expression of ATL2 transcripts were compared between breast tumors from The Cancer Genome Atlas (TCGA) and normal breast tissue from GTEX in the Xenabrowser database (https://xenabrowser.net/transcripts). The tumors are shown in pink and normal tissue in green. Expression is denoted as log2 TPM (transcripts per million). ATL2-1 (ENST00000378954.8, green) encodes the longest protein, 583 amino acids; ATL2-2 (ENST00000419554.6, orange) encodes 579 amino acids, and ATL2-3 (ENST00000406122.5, blue, top) encodes 413 amino acids. ATL2* (ENST00000477642.5, blue, bottom) appears to be an incomplete transcript as both 5´ and 3´ ends are missing. In this study, ATL2-1 and ATL2-2 refer to the same transcripts/proteins as in Crosby et al. (PMID: 34817557).

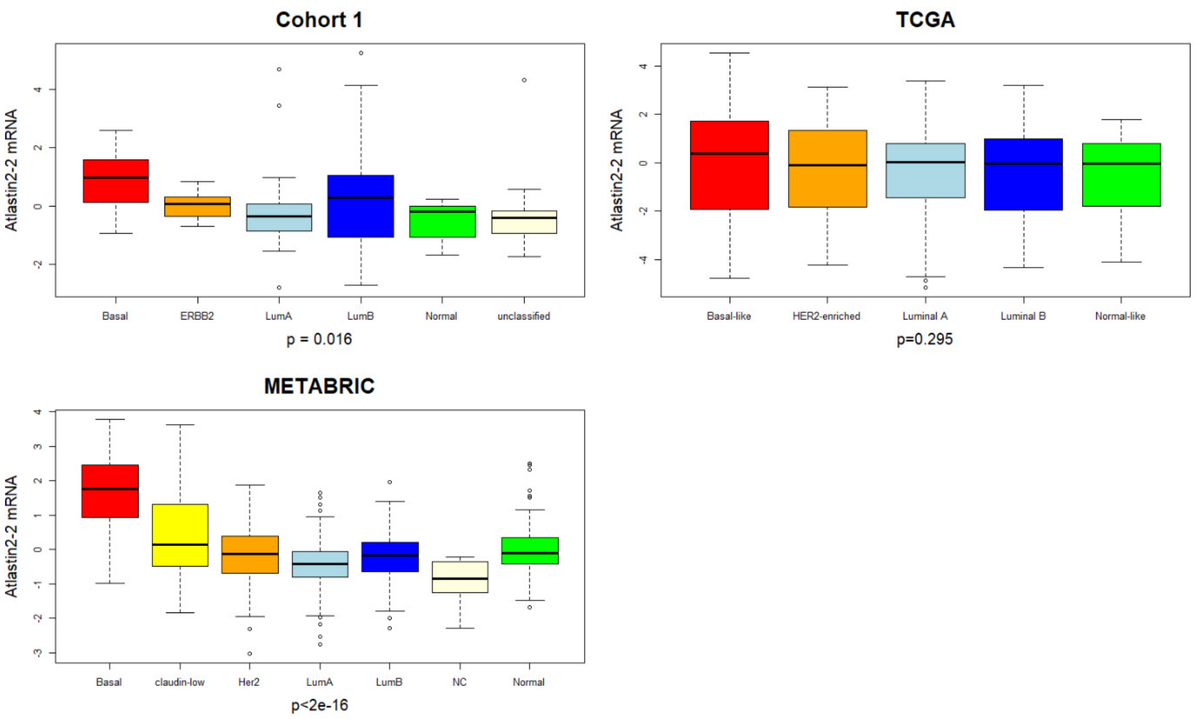

Figure S2. ATL2-2 expression levels in the molecular subtypes. A correlation analysis between ATL2-2 levels and molecular subtypes was performed in Cohort 1, TCGA and METABRIC. Tumors in Cohort 2 have not been classified into molecular subtypes. The box plot figures show the median values and distribution of ATL2-2 mRNA levels within each cohort according to molecular subtype. The number of samples in basal-like, Claudin-low, HER2/ERBB2, Luminal A, Luminal B, normal-like and unclassified/NC were in Cohort 1: 24, 0, 14, 39, 28, 12 and 11; TCGA: 140, 0, 62, 373, 149, 27 and 0; METABRIC: 198, 199, 220, 678, 461, 140, and 6. The difference in ATL2-2 expression levels between the molecular subtypes was calculated with one-way ANOVA. The p-values were Cohort 1: p=0.016; $p=4x10^{-5}$, TCGA: p=0.295, METABRIC: $p<2*10^{-16}$. The post-hoc Tukey test showed that the difference was between basal-like and the subtypes ERBB2, luminal A and normal in Cohort 1; between basal-like and luminal A in TCGA; and between basal-like and the subtypes Her2, luminal A and B and normal-like in METABRIC.

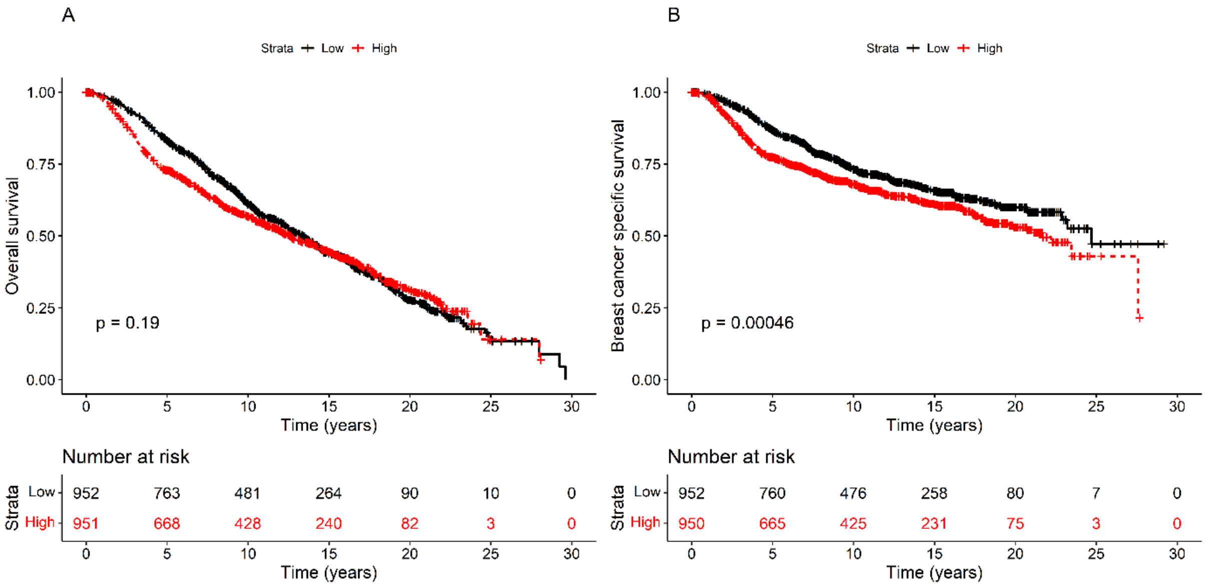

Figure S3. High ATL2-2 mRNA expression levels did not associate with BCSS in METABRIC cohort after correction for estrogen receptor status. Overall survival (OS) and breast cancer specific survival (BCSS) was analyzed in the whole METABRIC cohort. Patients were divided into two groups according to ATL2-2 mRNA expression in the breast tumors, low are expression levels below the median (black line) and high are expression levels above the median (red line). The BCSS log rank p-value was $4.6x10^{-4}$ but after correction for estrogen receptor status the log rank p-value was 0.076 indicating that the effect on survival was due to estrogen receptor status. The number of patients at risk at the indicated time point is shown in tables below the Kaplan-Meier graphs.

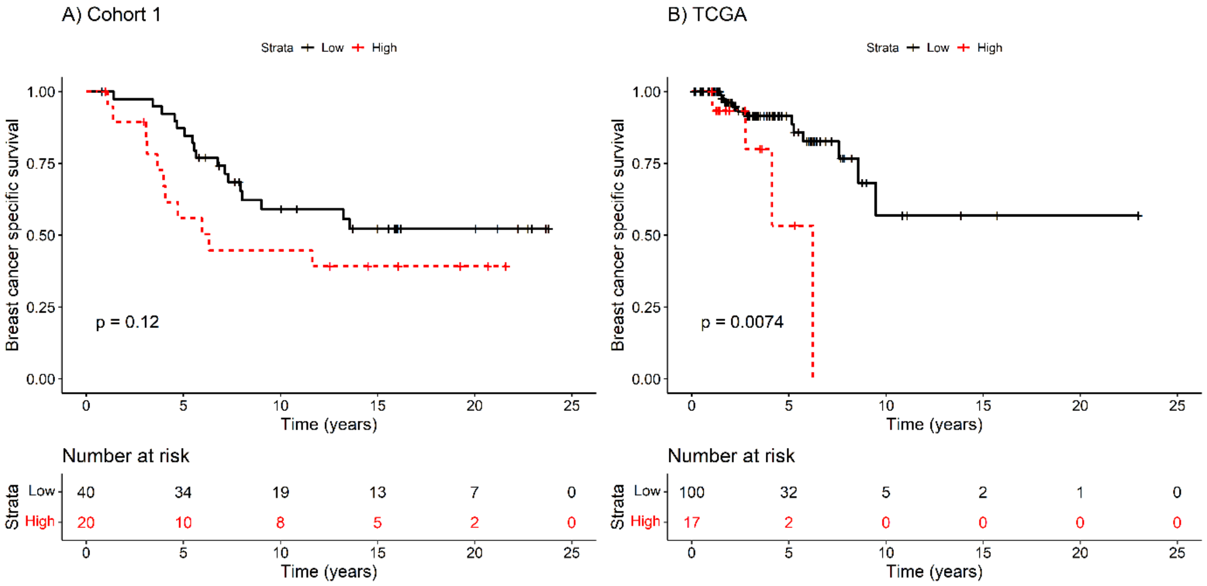

Figure S4. Cohort 1 and TCGA patients with high ATL2-2 mRNA levels have shorter BCSS in patients with estrogen receptor positive-luminal tumors. Breast cancer specific survival (BCSS) was analyzed in A) Cohort 1 and B) TCGA (patients diagnosed 1988-2005) in patients whose tumors expressed the estrogen receptor and were classified as luminal according to molecular subtyping. ATL2-2 mRNA values were divided based on the max stat function that finds the best division based on outcome. The log rank values are depicted in the graphs and below them in the tables are the number of patients at risk at the indicated time points.

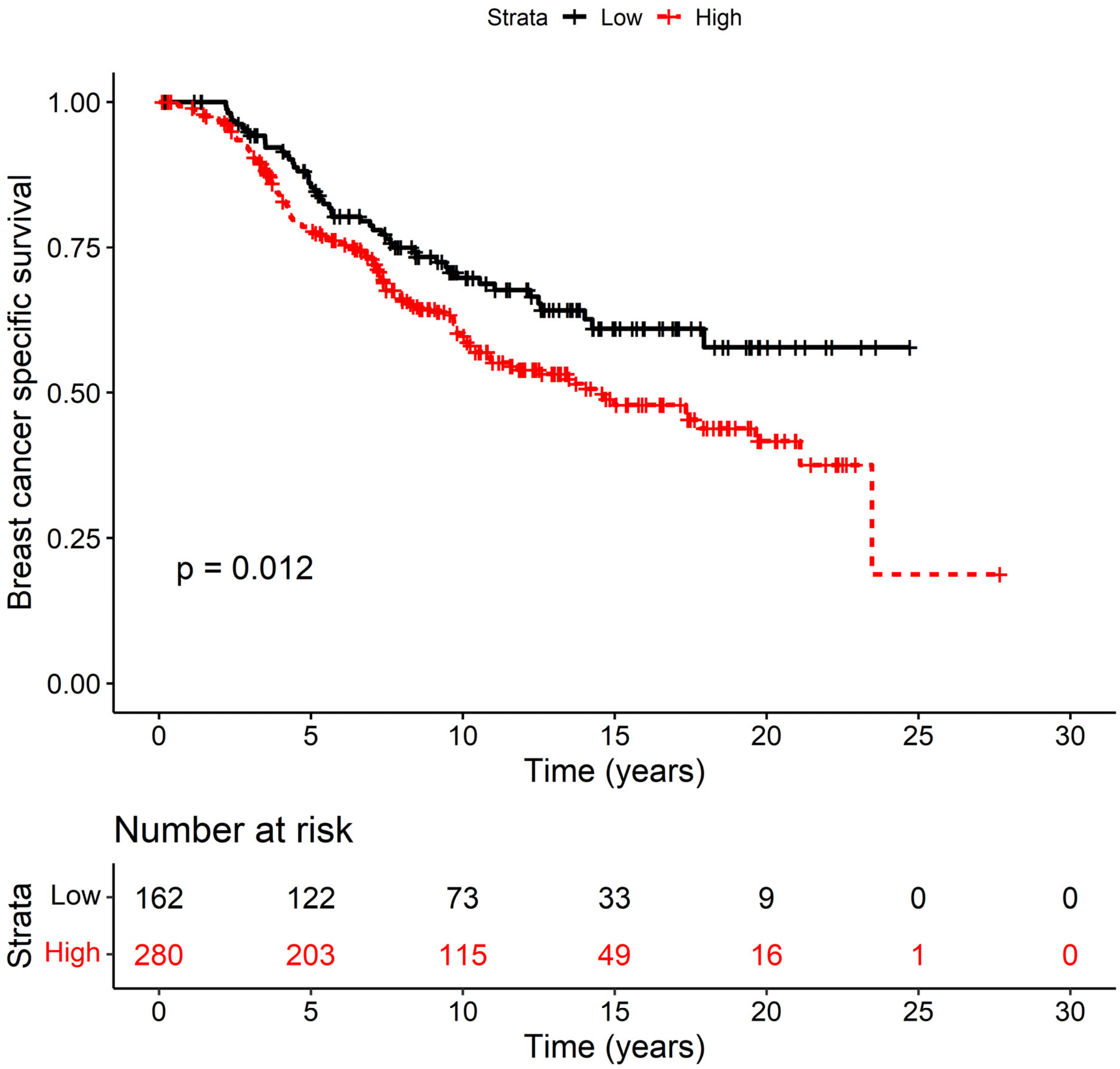

Figure S5. High ATL2-2 expression levels associated with shorter BCSS in patients with estrogen receptor positive-luminal B tumors. Breast cancer specific survival (BCSS) was analyzed in the METABRIC cohort in patients whose tumors expressed the estrogen receptor and were classified as luminal B according to molecular subtyping. The ATL2-2 mRNA values in the tumors were divided based on the max stat function that finds the best division based on outcome. In the low expressing group were 162 (black line) and 280 in the group expressing high ATL2-2 (red line). The log rank p-value was 0.012. The number of patients at risk at the indicated time point is shown in a table below the Kaplan-Meier graph. The HR was 1.524 (CI 1.096-2.118) prior to adjusting for confounding variables. Suppl Table 5 shows the hazard ratios (HR) and confidence interval (CI) from the Cox regression analysis prior to and after adjusting for confounding variables. Although the association of high ATL2-2 expression was reduced after correction for clinicopathological variables, the HR remained significant.
